# Fatal Clostridium septicum gas gangrene complicating ECMO: case report and review of literature

**DOI:** 10.1099/acmi.0.000825.v3

**Published:** 2024-08-05

**Authors:** Adrien Turban, Vincent Joussellin, Caroline Piau, Vincent Cattoir, Yoann Launey, Gabriel Eustache

**Affiliations:** 1 Department of Bacteriology, University Hospital of Rennes, 2 Rue Henri Le Guilloux, 35000 Rennes, France; 2 Critical Care Unit, Department of Anaesthesia, Critical Care and Perioperative Medicine, University Hospital of Rennes, Rennes, France; 3 UMR_S 1230 BRM, Inserm/University of Rennes, 2 Avenue du Pr. Léon Bernard, 35000 Rennes, France

**Keywords:** *Clostridium septicum*, ECMO complication, gas gangrene

## Abstract

*Clostridium septicum* gas gangrene is a severe and deadly infection caused by an anaerobic, spore-forming, Gram-positive bacillus. As previously described, two entities are observed: traumatic and spontaneous (or non-traumatic) forms. In this report, we aim to describe the case of a fulminant and ultimately fatal *C. septicum* myonecrosis occurring in a patient who was first admitted for refractory cardiac arrest and placed on veino-arterial extracorporeal membrane oxygenation (ECMO). Building upon prior studies that have documented cases of spontaneous gas gangrene caused by *C. septicum*, we provide an updated compilation, focusing on microbiological characteristics of *C. septicum*, along with the diagnostic and therapeutic challenges associated with spontaneous gas gangrene. Additionally, the specific clinical situation of our case illustrates the seriousness of this infectious complication that combined both spontaneous and traumatic gas gangrene risk factors. We thus, discuss the antibiotic coverage prior to the initiation of ECMO procedure.

## Data Summary

No data were generated during this research or are required for the work to be reproduced.

## Introduction

Extracorporeal membrane oxygenation (ECMO) is a final resort method employed in the treatment of patients experiencing severe acute respiratory distress syndrome, cardiogenic shock or refractory cardiac arrest. The substitution system enables blood oxygenation using an external oxygenation membrane. Blood is withdrawn from the human body via a venous catheter and then reintroduced, following oxygenation, through a second venous or arterial catheter, creating a venous-venous or venous-arterial circuit [1,2]. Despite significant progress in care and support of patient undergoing ECMO, mortality in intensive care units (ICUs) remains high: from 43 % in acute respiratory distress syndrome [3] to 61 % in cardiogenic shock [4]. In refractory cardiac arrest cases, the long-term survival and neurological prognosis associated with ECMO effectiveness remains uncertain [5].

Among complications associated with these devices, bleeding and hospital-acquired infections (HAIs) are both frequent and severe, and contribute to the ECMO’s high mortality [6]. HAI prevalence rates range from 10–12 % and are related to an increased risk of mortality, which varies from 38–63 % [1,7]. Among bacterial etiologies, coagulase-negative staphylococci (CoNS) are prevailing, followed by *Pseudomonas aeruginosa* (10.5 %), *Staphylococcus aureus* (9.4 %) and *Enterococcus* spp. (4 %). Notably, recent data suggest a growing significance of *Candida* spp., of which the implication may potentially surpass that of CoNS [8]. The clinical presentation of HAIs are mostly characterized by pulmonary lesions (56 %) and bacteremia or candidemia (26 %) [8,10]. Skin and soft-tissue infections are also described but remain scarce [8].

Gas gangrene (also called clostridial myonecrosis) is a rare acute infection disease that involved both subcutaneous and muscular tissues. It is mainly caused by *Clostridium* species such as *Clostridium perfringens, Clostridium septicum, Clostridium sordellii, Clostridium histolyticum* and *Clostridium novyi* [11,12]. It is composed of two clinical entities: 1) the traumatic gas gangrene, which represents about 85 % of cases, is caused by accidental (50 %) or surgical (35 %) traumas with the major injury leading to the invasion of soft tissues by clostridial spores through the wound (mostly from an environmental source) [13,14]; 2) the spontaneous (non-traumatic) gas gangrene, that represents about 15 % of cases, which occurs without any external injury and is often associated with gastrointestinal malignancy (61.5 %), other immunosuppression (malignant haematological disorder, chemotherapy or radiotherapy) or diabetes mellitus [12,13, 15,19]. *C. perfringens* is the major pathogen related to traumatic entities (80 %), while *C. septicum* is the main species involved in spontaneous cases [12,20,22]. Overall involvement of *C. septicum* in gas gangrene, whether spontaneous or not, is estimated at 20 % [23] and accounts for 1.3 % of all *Clostridium* infections [24].

In this case report, we describe the fatal case of a male patient who was first hospitalized for refractory cardiac arrest requiring venous-arterial ECMO, which subsequently led to a *C. septicum* gas gangrene. To our knowledge, this is the first case in the literature.

## Observation

**Fig. 1.**
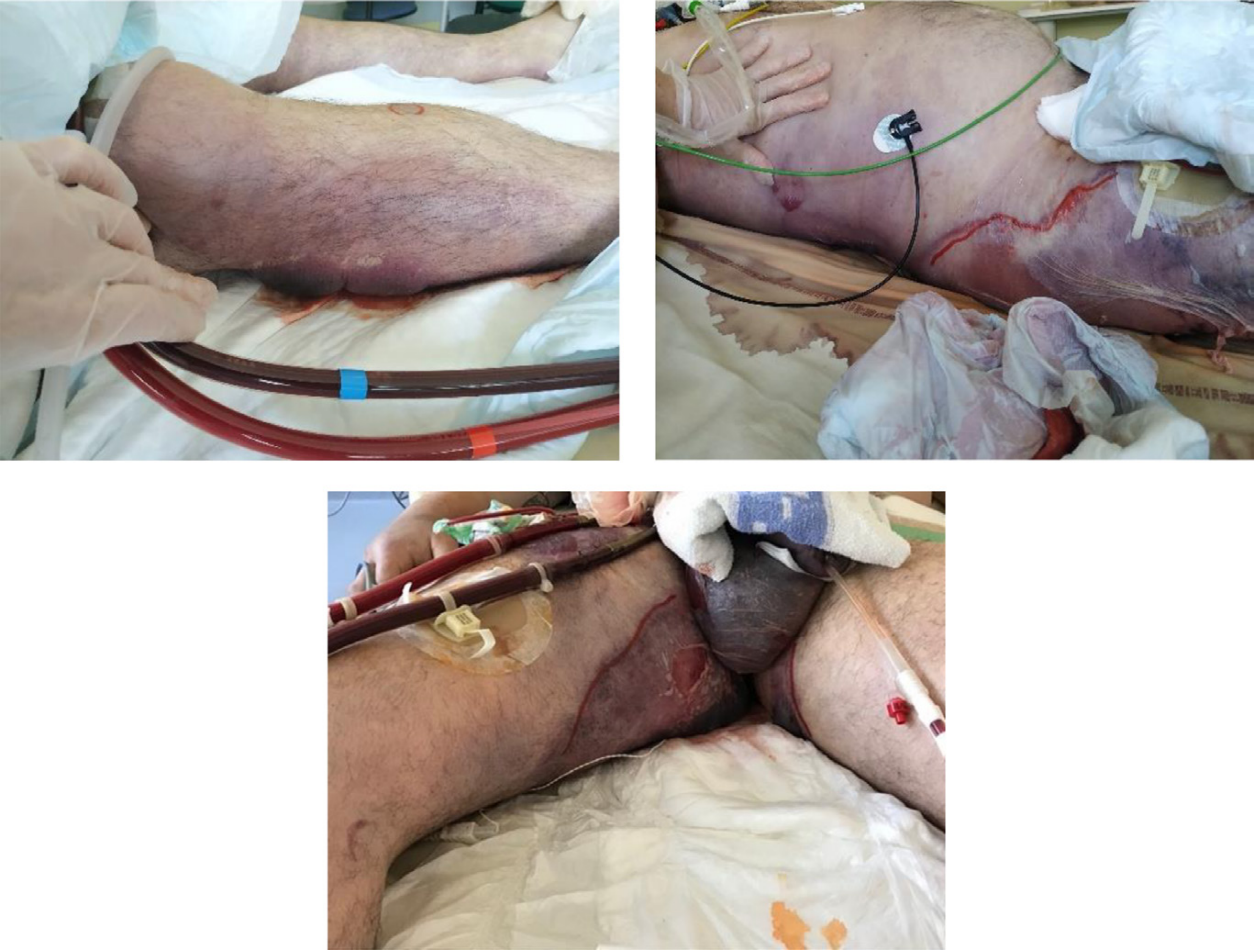
Images of the right leg, right flank and perineum.

We present the case of a 45-year-old male patient with no prior medical history, except for being overweight (BMI 28.9 kg m^−2^), who was hospitalized following a refractory cardiac arrest. The symptoms began 48 h before admission with the onset of epigastric pain and bronchial symptoms. He subsequently developed intermittent chest pain, described as burning, without radiation, along with pallor and sweating, prompting the initial call to emergency services. Cardiopulmonary arrest was confirmed by his partner at 04 : 45. Cardiopulmonary resuscitation was initiated immediately (no-flow <1 min). Medical team arrived at 5 : 08 and confirmed cardiopulmonary arrest due to ventricular fibrillation. Three external electrical shocks of 200 joules were delivered with no return to effective circulatory activity before intravenous administration of 300 mg of amiodarone and the first dose of epinephrine (1 mg) at 5 : 14. Orotracheal intubation was performed at 5 : 13, and the EtCO_2_ was 48 mmHg. The patient was transferred to the University Hospital of Rennes while receiving automated external chest compressions. During the transfer, six additional external electrical shocks of 200 joules were administered, along with a second dose of intravenous amiodarone (150 mg) and intravenous lidocaine (90 mg). Signs of life were observed with limb flexion in response to stimulation and eye opening upon request. Upon arrival at our clinical centre at 6 : 21, there was no spontaneous circulatory activity. Veino-arterial ECMO was initiated at 6 : 38, resulting in a 90 min low-flow period. The femoro-femoral veino-arterial ECMO was established through direct surgical access of the right scarpa. A reperfusion cannula was inserted into the right superficial femoral artery. ECMO flow was set at 5 l min^−1^ with a r.p.m. of 3900 min^−1^. The FmO_2_ was set to obtain a SpO_2_ ≥92 %. The correct position of cannula was confirmed by echocardiography and chest X-ray. No complications were reported. After haemodynamic stabilization, a coronary angiography revealed acute occlusion of the proximal and mid-right coronary artery. Thromboaspiration was followed by the placement of two active stents. Dual antiplatelet therapy with intravenous lysine acetylsalicylate (250 mg) and oral clopidogrel (600 mg) was initiated along with curative sodium heparin anticoagulation. Echocardiography showed diffuse akinesia, and sub-aortic valve systolic index was measured at 3.5 cm (with ECMO flow set at 5.0 l min^−1^). The initial evolution was marked by vasoplegia and hypovolemia due to post-resuscitation syndrome. The patient received 10 000 ml of crystalloid solution and norepinephrine up to 0.2 µg/kg min^−1^ in the first day of intensive care. Sedation and neuromuscular blockade initiated at ECMO implantation were discontinued after 24 h of targeted temperature management (temperature maintained between 35 and 37 °C). Significant signs of awakening were observed with eye opening and limb movements upon request. Thirty-six hours after admission to ICU, the patient presented haemodynamic deterioration. Echocardiographic showed a significant improvement in overall left ventricular systolic function, and the sub-aortic valve systolic index was measured at 9 cm (ECMO flow set at 5.5 l min^−1^). Norepinephrine was gradually increased up to 1.25 µg/kg min^−1^. Concurrently, severe hypoxemia (PaO_2_ 67 mmHg despite FiO_2_ 100 % and FmO_2_ 100 %) was observed. Chest X-ray revealed bilateral alveolo-interstitial opacities. Clinically, a unique purplish-violet lesion, 2 cm in size, was noted on the inner surface of the right leg. Empirical antibiotic therapy with a loading dose of 2 g of cefotaxime followed by a 6 g day^−1^ infusion and a 30 mg kg^−1^ dose of amikacin was initiated after blood cultures and endotracheal aspiration were performed. At 48 h after admission, epinephrine was added at a dose of 0.5 µg/kg min^−1^, along with an increase in norepinephrine to 2 µg/kg min^−1^. Within an hour, a large, extended, and haemorrhagic bullous detachment was observed from the right leg to the right flank and perineum (Fig. 1). Subsequently, subcutaneous crepitus was noted on the right calf.

**Fig. 2.**
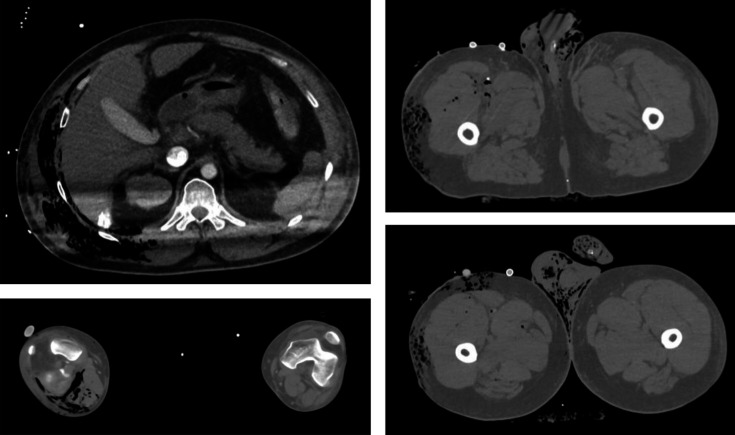
Pre-mortem CT images.

Hematologic tests then revealed anaemia with a haemoglobin level of 5.4 g dl^−1^ compared to 8.1 g dl^−1^ 2 h earlier. Massive transfusion with seven units of red blood cells, seven units of fresh frozen plasma, and one unit of platelets was initiated in case of haemorrhagic complications. The clinical course deteriorated further with refractory shock, and surgical intervention was deemed futile. A pre-mortem computed tomography (CT) scan revealed emphysematous infiltration of the subcutaneous soft tissues, primarily intramuscular, of the right postero-lateral abdominal wall, extending to the right iliopsoas muscle and the right leg, and the right pectoral muscle. There was extensive emphysematous infiltration of the subcutaneous fatty tissues of the right leg, scrotum, and penis (Fig. 2). Air bubbles were present in the venous network of the right lower limb, extending to the right femoral vein, the inferior vena cava, and the left renal vein. Microbiological culture of endotracheal aspiration revealed a polymorphic flora.

**Fig. 3.**
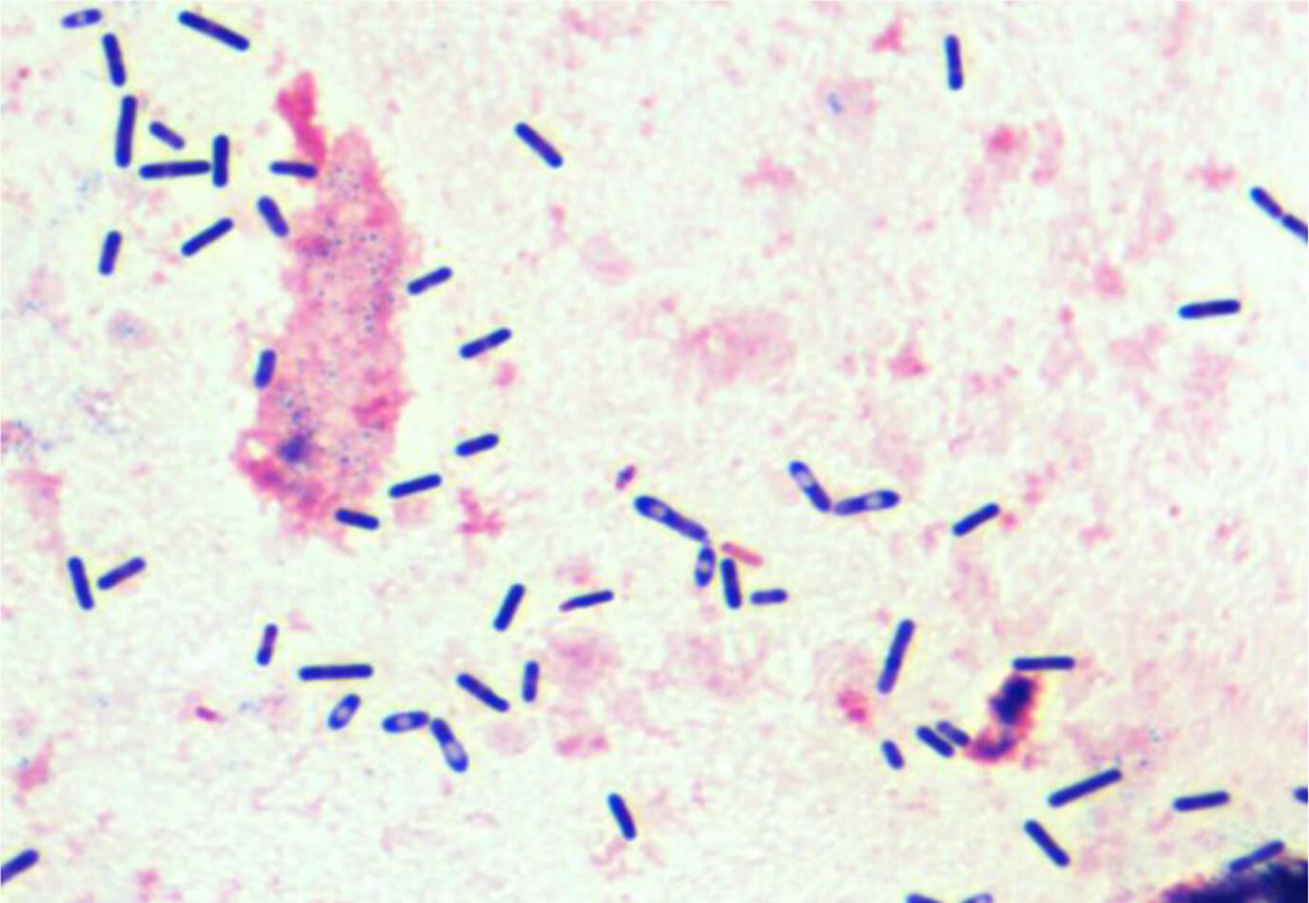
Blood culture showing Gram-positive, spore forming bacillus.

Blood cultures and liquid from haemorrhagic bullae were incubated in both aerobic and anaerobic blood culture bottles. The anaerobic cultures revealed the presence of a Gram-positive, spore-forming bacillus. These anaerobic bottles were then subcultured onto agar plates under anaerobic conditions. Colonies were identified by MALDI-TOF mass spectrometry (Bruker Daltonics, Germany), with both samples yielding positive for *C. septicum* (Fig. 3). Antibiotic susceptibility was determined using the reference disc-diffusion method, according to 2022 CA-SFM/EUCAST guidelines, with Brucella agar supplemented with 5 % sheep blood, 5 mg l^−1^ hemin, and 1 mg l^−1^ vitamin K. *C. septicum* was susceptible to all tested antibiotics: amoxicillin/clavulanate, piperacillin/tazobactam, imipenem, moxifloxacin, clindamycin, linezolid, vancomycin, and metronidazole (according to 2022 CA-SFM/EUCAST clinical breakpoints). The patient succumbed 58 h after hospital admission.

### Clostridium septicum

*C. septicum* is an opportunistic, anaerobic, spore-forming Gram-positive bacillus. These spores represent the resistance and dissemination form of *Clostridium* species. It is often found in the soil and in animal digestive tracts [17,23, 25]. Whether * C. septicum* is a normal commensal bacteria in the human gut remains unclear, however carriage frequency appears to be low [26].

#### Virulence and toxins

*C. septicum* exerts several important bacterial factors involved in its virulence. In contrast to other *Clostridium* species, such as * C. perfringens, C. septicum* displays an aerotolerant metabolism, thus explaining its ability to proliferate in healthy tissues, even in the absence of a strict anaerobic environment [27]. In a murine model, the bacterial load necessary to cause infection is 300-fold lower than with *C. perfringens,* and with only a few quantities of spores it can cause fatal disease in immunocompromised patients [27,28]. Like many flagellated bacteria, *C. septicum* has the ability to develop collective mass swarming behaviour, notably in nutrient-restricted conditions [29], participating to coordinate movements on surfaces, thereby facilitating tissue invasion [29]. Additionally, *C. septicum* demonstrates the capacity to form biofilms through several features like flagella or biofilm-associated proteins, showing positive correlation with antibiotic resistance and resulting in a worse prognosis [28]. *C. septicum* virulence relies mostly on the synthesis of a necrotic exotoxin called α-toxin which is coded by the *csa* gene [30,32]. It belongs to the aerolysin family, like the ε-toxin produced by *C. perfringens* and a toxin secreted by *Aeromonas hydrophila* [17,20, 25]. It is initially secreted as an inactive preprotoxin state, subsequently activated by various host proteases, resulting in oligomerization and formation of membrane pores leading to programmed cellular necrosis [17,20, 31, 33]. These membrane pores play a crucial role in tissue necrosis, leading to osmotic cell lysis and an intracellular influx of Ca^2+^ ions, initiating a signalling cascade resulting in programmed cell necrosis [34]. Additionally, α-toxin leads to decreased tissue perfusion, paving the way for anaerobic conditions to emerge. It also reduces the chemotactic response of neutrophilic polymorphonuclear leukocytes at the site of infection. This toxin induces an increase in capillary permeability, probably explaining frequent metastatic dissemination. Furthermore, α-toxin actively promotes the secretion of HMGB1, a protein that affects neutrophil-dependent antibacterial mechanisms and may participate in the development of a fulminant septic state [16,17, 20, 34,36]. *C. septicum* also produces other enzymes including β-toxin (DNAse), γ-toxin (hyalurodinase) or δ-toxin (hemolysin), that may contribute to an increased capillary permeability, myonecrosis and toxin dissemination [28]. *C. septicum* displays many other virulence factors as neuraminidase, protease, sialidase or fibrinolysin which may contribute to systemic dissemination through tissue damages [17,28, 37, 38].

#### Antibiotics susceptibility

Data on *C. septicum* remains limited in the literature. It is usually susceptible to penicillin or metronidazole [12,39,41]. In contrast to *C. butyricum, C. clostridioforme, C. ramosum* or *C. innoccum,* no β-lactamase has been described in *C. septicum*. However, resistance to other antibiotics has been described. In a study involving 78 strains, 24.3 % showed resistance to vancomycin [28]. Moreover, resistance to clindamycin appears to be more prevalent, as resistant strains are observed in multiple studies (ranging from 34.6–69 % of tested isolates) [28,41, 42].

### *C. septicum* gas gangrene

#### Spontaneous gas gangrene

##### Pathophysiology

Gas gangrene is a deadly disease, showing high mortality (67–100 %), especially in the first hours [12,17]. Spontaneous gas gangrene occurs without any major external trauma. Most of these spontaneous necrotic infections are caused by *C. septicum,* and several host factors, especially immunosuppression conditions, favour its development [17]. In 2017, Srivastava *et al*. [17] showed that in patients with spontaneous gas gangrene, 71 % had an underlying malignancy, mostly digestive [17]. The principal hypothesis explaining the high correlation between spontaneous gas gangrene and cancer or immunosuppression lies on the probable association between a transient carriage and host factors. This may result in increased colonization and/or higher susceptibility to infection [17]. Tissue destruction generated by *C. septicum* virulence factors maintains an anaerobic environment, allowing bacterial multiplication associated with gas production leading to clinical crepitus, characteristic of clostridial gas gangrene [35,43].

##### Clinical presentation

Clinical features are characterized by progressively increasing muscular pain, often disproportionate regarding clinical impact and mostly affecting the limbs [17,18, 27]. Clinical deterioration appears to be faster than with *C. perfringens* [27] and rapidly leads to apparition of haemorrhagic bullae, as observed in our case, change in skin coloration and the emergence of tissular necrosis [17]. It is important to note that gas crepitus is not systematically present at diagnosis, and may occur in late stages of the infection [17,44,46]. Timeline between infection and necrosis can be fulminant and can last between 6 to 48 h [18,35, 44, 47]. Progression to septic shock, multi-organ dysfunction and death often occurs rapidly (< 24 h), and mortality rates are higher in myonecrosis secondary to *C. septicum* (79 %) than in those caused by *C. perfringens* (32 %) [35].

##### Diagnosis

Diagnosis of spontaneous gas gangrene can be difficult, mainly due to the lack of a clear entry point, the non-specific initial symptomatology and the rapid evolution toward septic shock. Mean time of incubation is between 12 to 24 h and myonecrosis can extend as fast as 2 cm per hour [17,48]. The diagnosis relies on clinical criteria, as imaging and microbiological samples must not delay medical care [44]. Disparity between severe pain and clinical presentation, rapid haemodynamic deterioration, onset of haemorrhagic bullae or skin necrosis should raise suspicion of necrotic involvement of soft tissues [43,49]. The input of imaging must be restricted to situations where clinical presentation is unclear. However, it should not delay any surgical or medical care [43,47]. X-ray radiography could contribute to faster identification of gas into tissue [35], but this technique shows low sensitivity [44,50], as does ultrasound imaging [43,47]. CT scan has the ability to characterize tissue impairment, reveal gas or evaluate infection evolution. Finally, MRI is an effective method to identify soft tissue necrosis, but access times are often incompatible with emergency situations and CT scan is generally preferred [45,47, 51]. Surgery is also crucial in diagnosis, allowing confirmation of tissular necrosis and microbiological sampling. The identification of spore-forming Gram-positive bacilli should prompt to antibiotics stewardship [17,18, 45].

##### Therapeutics

The management of these gas gangrenes relies on a medical-surgical approach, where the debridement of necrotic tissues remains the absolute emergency [17,18, 46]. Survival is increased in patients for whom surgery occurs within the first 24 h post-admission, even more within 6 h [46,50]. Medical approach includes probabilistic antibiotic therapy as Infectious Diseases Society of America (IDSA) recommends the use of a large-spectrum association of molecules: vancomycin +piperacillin/tazobactam or a carbapenem. A combination of intravenous penicillin (2–4 million unit/4–6 h) and clindamycin (600–900 mg/8 h), due to its anti-toxin activity, is therefore recommended when *Clostridium* spp. is identified [18,46, 50, 52, 53]. The place of hyperbaric oxygen in management of gas gangrene remains discussed since the lack of animal and clinical data does not allow for its clear role in those infections. Furthermore, as *C. septicum* exhibits better aerotolerance, hyperbaric oxygenation is actually not recommended and should not delay surgical intervention [17,18, 43, 46, 50, 52]. Finally, as underlying diseases are often associated with spontaneous gas gangrene, investigation must be done when patient’s condition allows for it.

##### Review of *C. septicum* spontaneous gas gangrene literature

In 2017, the review of Srivastava *et al*. [17] focused on 94 *C*. *septicum* spontaneous gas gangrene cases, from 1956 to 2016. To complement the existing data, we carried out an extensive PubMed research for all cases using the terms ‘gas gangrene’, ‘*clostridium septicum*’ and ‘clostridial myonecrosis’. Through 2017 to 2022, we identified 21 cases in English literature on the basis of title and abstracts [23,54,73] (Table 1). Our review revealed an overall mortality rate of 71 %, and underlying diseases, whether known or not, were present in most cases, with a notable association with digestive malignancies (Table 2). The median age was 64 years, with two cases occurring in paediatric population. In the present review, diabetes mellitus was described in only five cases (24 %) (Table 2), which is less than previous data (41 %) [17]. Implications of diabetes may be related to decreased phagocytosis and chemotactic activity in patients with glycaemic dysregulation diseases [17]. During hospitalization, 71 % of the patients exhibited change in skin coloration (discolouration, erythema, bruising), 43 % had oedema and 43 % had fever. Every single patient experienced important pain and crepitus was found in 66 % (Table 3). Cumulative data of both ours and a previous study [17] shows that a combination of surgical and medical management led to a decreased mortality rate (57 %) compared to cases where only antibiotics or no medical care was considered (86–100 %), emphasizing the importance of multidisciplinary intervention (Table 4). Data available on antibiotics used revealed the frequent association between clindamycin and a β-lactamine (penicillin, piperacillin/tazobactam, cephalosporin or meropenem) (Table 1).

**Table 1.** Summary of features of *C. septicum* spontaneous gas gangrene cases between 2017 and 2022

Years	Author	Age	Sex	Treatment	Antibiotics	Underlying disease	Outcomes
2017	Abdulkareem *et al*.	69	M	Surgery+antibiotics	Piperacillin/tazobactam+vancomycin	Chronic lymphocytic leukaemia	Survival
2017	Contou *et al*.	25	M	Surgery+antibiotics	Amoxicillin/clavulanic acid+clindamycin+amikacin	Leucopenia	Death
2017	Cullinane *et al*.	69	M	Surgery+antibiotics	Vancomycin+clindamycin+metronidazole	Diabetes and cecal tumour	Survival
2017	Engen *et al*.	3	F	No treatment	No treatment	Hemolytic uremic syndrome	Death
2017	Mytinger *et* Kraai	63	M	No treatment	No treatment	Rheumatoid arthritis	Death
2018	Thompson *et al*.	57	M	Surgery+antibiotics	Not specified	Prostate cancer	Death
2019	Hussain *et al*.	23	F	Surgery+antibiotics	Piperacillin/tazobactam+clindamycin and penicillin	Infectious colitis	Survival
2019	Saunders *et al*.	76	F	Surgery+antibiotics	Not specified	Diabetes, intestinal adenocarcinoma	Death
2019	Senghaas *et al*.	72	F	Surgery+antibiotics	Not specified	Breast cancer	Survival
2020	Chen *et al*.	33	M	Surgery+antibiotics	Not specified	Colorectal cancer	Death
2020	Grey *et al*.	56	F	Surgery+antibiotics	Penicillin+clindamycin+vancomycin	Breast cancer	Death
2020	Wongboosin *et al*.	64	M	No treatment	No treatment	Schizophrenia	Death
2020	Saiyed *et al*.	57	F	Antibiotics	Not specified	Mantle cell lymphoma	Death
2021	Ben Ismail *et al*.	58	F	Surgery+antibiotics	Not specified	Diabetes	Death
2021	Stoddard *et al*.	67	M	Antibiotics	Meropenem	Diabetes	Death
2021	Parmar *et al*.	14	F	Surgery+antibiotics	Ceftazidime+metronidazole and penicillin	Burkitt lymphoma	Survival
2021	Sivasubramanian *et al*.	83	M	Antibiotics	Vancomycin+meropenem+clindamycin	Diabetes, colorectal cancer	Death
2022	Bickerton *et al*.	76	F	Surgery+antibiotics	Piperacillin/tazobactam+clindamycin	Colorectal cancer	Death
2022	Hechter *et al*.	77	F	Surgery+antibiotics	Piperacillin/tazobactam+vancomycin	Colorectal cancer	Death
2022	Van Asbroeck *et al*.	84	F	Surgery+antibiotics	Amoxicillin/clavulanic acid	Colorectal cancer	Death
2022	Slezak *et al*.	86	F	Surgery+antibiotics	Ampicillin/sulbactam+clindamycin+metronidazole	Gastrointestinal adenocarcinoma	Survival

**Table 2.** Underlying disease, treatment and blood cultures results of *C. septicum* spontaneous gas gangrene cases between 2017 and 2022

	N (%)	Death (%)
Cases (n)	21 (100)	71
Immunosuppression	15 (71)	66
Gastrointestinal malignancy	8 (53)	75
Haematological	4 (26)	50
Other immunosuppression	3 (20)	66
Diabetes*	5 (24)	80
Treatments		
Surgery+antibiotics	15 (71)	60
Antibiotics alone	3 (14)	100
No treatment	3 (14)	100
Blood cultures		
Positive to *C. septicum*	13 (62)	85
Negative	1 (5)	0
Not specified	7 (33)	57

*Diabetes can be associated with immunosuppressive underlying condition.

### Other *C. septicum* infections

Current literature on *C. septicum* infections preferentially refers to spontaneous gas gangrene. Few cases describe traumatic presentation, following surgical intervention [49,68, 74,76], after accidental trauma [77,78] or secondary to insulin injection [79], with fatal outcomes in the majority of reported cases. *C. septicum* is also implicated in other clinical presentations: vascular infections (aortitis [80], aneurysms [24]), endophthalmitis [81], abscesses [82] or even meningitidis [83].

## Discussion

To our knowledge, this is the first case of *C. septicum* gas gangrene following veino-arterial ECMO cannulation. In our report, the source of infection appears to be related to the ECMO implantation site. However, an endogenous origin cannot be ruled out, as low-flow can favour anaerobic conditions, digestive ischemia and bacterial translocation. The rapid and fatal progression of this case raises the question about antibiotic prophylaxis of such life-saving surgical interventions. Indeed, the timing of ECMO implantation is an integral part of low-flow and justifies a need for speed. Despite implantation in a dedicated medical environment, disinfection and demarcation of the operative site with sterile drapes, early infections can still result from inadequate asepsis. Furthermore, the high risk of bacterial infection in the early phase of ECMO implantation, associated with the potential of bacterial translocation due to prolonged no-flow (particularly splanchnic) in the specific case of refractory cardiac arrest could justify antibiotic prophylaxis. Current recommendations (ELSO, Extracorporeal Life Support Organization) do not advocate for antibiotic prophylaxis for these procedures [84]. However, if antibiotic coverage is initiated, it is not recommended to extend it beyond 48 h [84]. Despite the lack of recommendations, 50–68 % of teams still use antibiotic prophylaxis. The most common antibiotics used are cephalosporins, penicillin, vancomycin or aminoglycosides [1]. Notably, there is no clinical data available on the efficacy of these protocols and administration prior to ECMO implantation does not appear to be associated with a decreased infection risk [1].

## Conclusion

Gas gangrene due to *C. septicum* is extremely serious disease, often fatal, and has never been described in the context of extracorporeal circulation. Such an invasive procedure is considered as ‘clean’ procedure (Altemeier class 1) for which no antibiotic is currently recommended. However, in the context of cardiac arrest, as presented in our case, several factors may explain the development of this *C. septicum* gas gangrene. The fatal outcome of this case could raise the question of using anti-clostridial antibiotics in the coverage of ECMO intervention, but the scarcity of described cases and current data do not allow the drawing of any conclusions and would require further studies.

**Table 3.** Summary of clinical features during hospitalization

Symptoms (*N*=21)	N	%
Pain	21	100
Fever/chills	9	43
Gastrointestinal disorders	6	28
Hypotension	15	71
Tachycardia	16	76
Septic shock*	11	52
Swelling/oedema	9	43
Skin involvement (discolouration, erythema, bruising)	15	71
Crepitus	14	66

*Patient with septic shock were included in both hypotension and tachycardia criteria.

**Table 4.** Cumulative data on *C. septicum* spontaneous gas gangrene following different treatment management

	Srivastava *et al*.		2017–2022		Cumulative	
	N (%)	Death (%)	N (%)	Death (%)	N (%)	Death (%)
Cases	94	67	21	71	115	68
Treatment	74*		21*		95*	
Surgery+antibiotics	54 (73)	56	15 (71)	60	69 (73)	57
Antibiotics alone	18 (24)	83	3 (14)	100	21 (22)	86
No treatment	2 (3)	100	3 (14)	100	5 (5)	100

*Only cases where such information was available were included.

## Abbreviations

BMI: body mass index
CA-SFM/EUCAST: Antibiogram Committee of the French Microbiology Society / European committee on antimicrobial susceptibility testing
CoNS: coagulase-negative staphylococci
CT: computed tomography
DNAse: deoxyribonuclease
ECMO: extracorporeal membrane oxygenation
EtCO2: end-tidal carbon dioxide
FiO2: fraction of inspired oxygen
FmO2: membrane fraction of oxygen
HAI: hospital-acquired infection
MALDI-TOF: matrix assisted laser desorption Ionization - time of flight
PaO2: arterial oxygen tension
r.p.m.: revolution per minute

## References

[1] Biffi S, Di Bella S, Scaravilli V, Peri AM, Grasselli G (2017). Infections during extracorporeal membrane oxygenation: epidemiology, risk factors, pathogenesis and prevention. Int J Antimicrob Agents.

[2] Gajkowski EF, Herrera G, Hatton L, Velia Antonini M, Vercaemst L (2022). ELSO guidelines for adult and pediatric extracorporeal membrane oxygenation circuits. ASAIO J.

[3] Schmidt M, Bailey M, Sheldrake J, Hodgson C, Aubron C (2014). Predicting survival after extracorporeal membrane oxygenation for severe acute respiratory failure. The respiratory extracorporeal membrane oxygenation survival prediction (RESP) score. Am J Respir Crit Care Med.

[4] Paden ML, Conrad SA, Rycus PT, Thiagarajan RR, Registry E (2013). Extracorporeal life support organization registry report 2012. ASAIO J.

[5] Belohlavek J, Smalcova J, Rob D, Franek O, Smid O (2022). Effect of intra-arrest transport, extracorporeal cardiopulmonary resuscitation, and immediate invasive assessment and treatment on functional neurologic outcome in refractory out-of-hospital cardiac arrest: a randomized clinical trial. JAMA.

[6] Zangrillo A, Landoni G, Biondi-Zoccai G, Greco M, Greco T (2013). A meta-analysis of complications and mortality of extracorporeal membrane oxygenation. Crit Care Resusc.

[7] Bizzarro MJ, Conrad SA, Kaufman DA, Rycus P (2011). Extracorporeal life support organization task force on infections, extracorporeal membrane oxygenation. Infections acquired during extracorporeal membrane oxygenation in neonates, children, and adults. Pediatr Crit Care Med Mai.

[8] Gomez F, Veita J, Laudanski K (2022). Antibiotics and ECMO in the adult population-persistent challenges and practical guides. Antibiotics.

[9] Schmidt M, Bréchot N, Hariri S, Guiguet M, Luyt CE (2012). Nosocomial infections in adult cardiogenic shock patients supported by venoarterial extracorporeal membrane oxygenation. Clin Infect Dis.

[10] Sun H-Y, Ko W-J, Tsai P-R, Sun C-C, Chang Y-Y (2010). Infections occurring during extracorporeal membrane oxygenation use in adult patients. J Thorac Cardiovasc Surg.

[11] Mallozzi M, Viswanathan VK, Vedantam G (2010). Spore-forming *Bacilli* and *Clostridia* in human disease. Future Microbiol.

[12] Stevens DL, Aldape MJ, Bryant AE (2012). Life-threatening clostridial infections. Anaerobe.

[13] Gnerlich JL, Ritter JH, Kirby JP, Mazuski JE (2011). Simultaneous necrotizing soft tissue infection and colonic necrosis caused by *Clostridium septicum*. Surg Infect.

[14] Hamid S, Gadré A, Fornander L, Sjöwall J, Muhrbeck M (2023). *Clostridium septicum* myonecrosis following gardening: a case report. Int J Surg Case Rep.

[15] Corredoira J, Grau I, Garcia-Rodriguez JF, García-País MJ, Rabuñal R (2017). Colorectal neoplasm in cases of *Clostridium septicum* and *Streptococcus gallolyticus* subsp. gallolyticus bacteraemia. Eur J Intern Med.

[16] Dahmus JD, Kotler DL, Kastenberg DM, Kistler CA (2018). The gut microbiome and colorectal cancer: a review of bacterial pathogenesis. J Gastrointest Oncol.

[17] Srivastava I, Aldape MJ, Bryant AE, Stevens DL (2017). Spontaneous *C.septicum* gas gangrene: a literature review. Anaerobe.

[18] Stevens DL, Bryant AE (2017). Necrotizing soft-tissue infections. N Engl J Med.

[19] Yildiz T, Gündeş S, Willke A, Solak M, Toker K (2006). Spontaneous, nontraumatic gas gangrene due to *Clostridium perfringens*. Int J Infect Dis.

[20] Nagahama M, Takehara M, Rood JI (2019). Histotoxic clostridial infections. Microbiol Spectr.

[21] Laupland KB, Edwards F, Furuya-Kanamori L, Paterson DL, Harris PNA (2023). Bloodstream infection and colorectal cancer risk in Queensland Australia, 2000-2019. Am J Med.

[22] Justesen US, Nielsen SL, Jensen TG, Dessau RB, Møller JK (2022). Bacteremia with anaerobic bacteria and association with colorectal cancer: a population-based cohort study. Clin Infect Dis.

[23] Van Asbroeck E, Vasileiadou O, De Laere S, Van Hedent E, Devue K (2022). *Clostridium myonecrosis* - a rare and underdiagnosed condition in the elderly: a case with severe skipping lesions and an overview of treatment guidelines. Int J Emerg Med.

[24] Alimi Y, Sosin M, Borsinger TM, Garrett JR, Salameh JR (2017). Implications of *Clostridium septicum* in vascular surgery: a case report and outcomes literature review. Ann Vasc Surg.

[25] Alves MLF, Ferreira MRA, Donassolo RA, Rodrigues RR, Conceição FR (2021). *Clostridium septicum*: a review in the light of alpha-toxin and development of vaccines. Vaccine.

[26] Kopliku FA, Schubert AM, Mogle J, Schloss PD, Young VB (2015). Low prevalence of *Clostridium septicum* fecal carriage in an adult population. Anaerobe.

[27] Stevens DL, Musher DM, Watson DA, Eddy H, Hamill RJ (1990). Spontaneous, nontraumatic gangrene due to *Clostridium septicum*. Clin Infect Dis.

[28] Kuzma J, Zavala-Meneses SG, Skultety L, Chmelar D, Ficík J (2023). Antibiotic resistance and biofilm-forming ability of α-toxin-positive *Clostridium septicum* isolates worsen patient prognosis. APMIS.

[29] Macfarlane S, Hopkins MJ, Macfarlane GT (2001). Toxin synthesis and mucin breakdown are related to swarming phenomenon in *Clostridium septicum*. Infect Immun.

[30] Kennedy CL, Krejany EO, Young LF, O’Connor JR, Awad MM (2005). The alpha-toxin of *Clostridium septicum* is essential for virulence. Mol Microbiol.

[31] Knapp O, Maier E, Mkaddem SB, Benz R, Bens M (2010). *Clostridium septicum* alpha-toxin forms pores and induces rapid cell necrosis. Toxicon.

[32] Popoff MR, Bouvet P (2009). Clostridial toxins. Future Microbiol.

[33] Melton JA, Parker MW, Rossjohn J, Buckley JT, Tweten RK (2004). The identification and structure of the membrane-spanning domain of the *Clostridium septicum* alpha toxin. J Biol Chem.

[34] Kennedy CL, Smith DJ, Lyras D, Chakravorty A, Rood JI (2009). Programmed cellular necrosis mediated by the pore-forming alpha-toxin from *Clostridium septicum*. PLoS Pathog.

[35] Smith-Slatas CL, Bourque M, Salazar JC (2006). *Clostridium septicum* infections in children: a case report and review of the literature. Pediatrics.

[36] Grégoire M, Tadié J-M, Uhel F, Gacouin A, Piau C (2017). Frontline science: HMGB1 induces neutrophil dysfunction in experimental sepsis and in patients who survive septic shock. J Leukoc Biol.

[37] Thomas P, Abdel-Glil MY, Subbaiyan A, Busch A, Eichhorn I (2021). First comparative analysis of *Clostridium septicum* genomes provides insights into the taxonomy, species genetic diversity, and virulence related to gas gangrene. Front Microbiol.

[38] Tran HA, Myint E (2007). Fulminant *Clostridium septicum* myonecrosis in well controlled diabetes: a case report. J Med Case Rep.

[39] Aldape MJ, Bayer CR, Rice SN, Bryant AE, Stevens DL (2018). Comparative efficacy of antibiotics in treating experimental *Clostridium septicum* infection. Int J Antimicrob Agents.

[40] Gabay EL, Rolfe RD, Finegold SM (1981). Susceptibility of *Clostridium septicum* to 23 antimicrobial agents. Antimicrob Agents Chemother.

[41] Sárvári KP, Schoblocher D (2020). The antibiotic susceptibility pattern of gas gangrene-forming *Clostridium* spp. clinical isolates from South-Eastern Hungary. Infect Dis.

[42] Leal J, Gregson DB, Ross T, Church DL, Laupland KB (2008). Epidemiology of *Clostridium* species bacteremia in Calgary, Canada, 2000–2006. J Infect.

[43] Bonne SL, Kadri SS (2017). Evaluation and management of necrotizing soft tissue infections. Infect Dis Clin North Am.

[44] Cline KA, Turnbull TL (1985). Clostridial myonecrosis. Ann Emerg Med.

[45] Hakkarainen TW, Kopari NM, Pham TN, Evans HL (2014). Necrotizing soft tissue infections: review and current concepts in treatment, systems of care, and outcomes. Curr Probl Surg.

[46] Peetermans M, de Prost N, Eckmann C, Norrby-Teglund A, Skrede S (2020). Necrotizing skin and soft-tissue infections in the intensive care unit. Clin Microbiol Infect.

[47] Runer A, Schneider F, Mayr R, Dammerer D, Roth T (2021). Blistering of the entire lower limb after knee arthroscopy: benign subcutaneous emphysema, gas gangrene or necrotizing fasciitis? A case report and review of the literature. Trauma Case Rep.

[48] Leiblein M, Wagner N, Adam EH, Frank J, Marzi I (2020). Clostridial gas gangrene - a rare but deadly infection: case series and comparison to other necrotizing soft tissue infections. Orthop Surg.

[49] van Samkar G, van der Hoeven J, Hollmann MW, Goslings JC (2009). Lethal complication after abdominal wall reduction. J Plastic Reconstr Aesth Surg.

[50] Stevens DL, Bisno AL, Chambers HF, Dellinger EP, Goldstein EJC (2014). Practice guidelines for the diagnosis and management of skin and soft tissue infections: 2014 update by the infectious diseases society of America. Clin Infect Dis.

[51] Goldstein EJC, Anaya DA, Dellinger EP (2007). Necrotizing soft-tissue infection: diagnosis and management. Clin Infect Dis.

[52] Allaw F, Wehbe S, Kanj SS (2024). Necrotizing fasciitis: an update on epidemiology, diagnostic methods, and treatment. Curr Opin Infect Dis.

[53] Stevens DL, Bryant AE, Hackett SP (1995). Antibiotic effects on bacterial viability, toxin production, and host response. Clin Infect Dis.

[54] Abdulkareem A, D’Souza RS, Shogbesan O, Donato A (2017). A case of rituximab-induced necrotizing fasciitis and a review of the literature. Case Rep Hematol.

[55] Ben Ismail I, Hammami N, Hakim Z, Rebii S, Zoghlami A (2021). Clostridial abdominal wall gas gangrene secondary to sigmoid cancer perforation. ANZ J Surg.

[56] Bickerton S, Awopetu A, Abood A, Lee H, Lane T (2022). Atraumatic *Clostridium septicum* myonecrosis presenting as upper limb ischaemia in a patient with undiagnosed bowel cancer. Ann R Coll Surg Engl.

[57] Chen LL, Tayban K, Caravanos C, Shaz D, Halpern NA (2020). Necrotizing fasciitis associated with malignancy. J Am Assoc Nurse Pract.

[58] Contou D, Lecronier M, Bitot V, Hersant B, Zakine A (2017). Fatal *Clostridium septicum* multifocal myonecrosis in a previously healthy 25-year-old man: role of NSAIDs?. Med Mal Infect.

[59] Cullinane C, Earley H, Tormey S (2017). Deadly combination: *Clostridium septicum* and colorectal malignancy. BMJ Case Rep.

[60] Engen RM, Killien EY, Davis JL, Symons JM, Hartmann SM (2017). *C septicum* complicating hemolytic uremic syndrome: survival without surgical intervention. Pediatrics.

[61] Gray KM, Padilla PL, Sparks B, Dziewulski P (2020). Distant myonecrosis by atraumatic *Clostridium septicum* infection in a patient with metastatic breast cancer. IDCases.

[62] Hechter S, Patel V, Bommu VJL, Patel P, Ao X (2022). Necrotizing fasciitis: a life-threatening infection due to *Clostridium* species. Cureus.

[63] Hussain C, Ball MK, McGwire BS (2019). Multidrug-resistant bovine Salmonellosis predisposing for severe human *Clostridial myonecrosis*. Am J Case Rep.

[64] Mytinger A, Kraai EP (2017). A man with severe back pain. Ann Emerg Med.

[65] Parmar P, Feder J, Pham-Huy A (2021). *Clostridium septicum* myonecrosis in a pediatric patient with a self-reported penicillin allergy. J Assoc Med Microbiol Infect Dis Can.

[66] Saunders RN, Hayakawa E, Gibson CJ, Chapman AJ (2019). *Clostridium septicum* myonecrosis secondary to an occult small bowel adenocarcinoma. J Gastrointest Canc.

[67] Senghaas A, Kremer T, Schmidt VJ, Harhaus L, Hirche C (2019). Sliding free transverse rectus abdominis myocutaneous flap for closure of A massive abdominal wall defect: a case report. Microsurgery.

[68] Sivasubramanian G (2021). Rapidly progressive and fatal distant spontaneous gas gangrene due to *Clostridium septicum* after biopsy of malignant cecal mass. IDCases.

[69] Thompson KM, Kruse BT, Hedges MAS (2018). Atraumatic *Clostridial myonecrosis* in an immunocompromised host. J Emerg Med.

[70] Wongboonsin J, Duran A, Johnson JR (2020). Infectious Pneumorachis due to *Clostridium septicum*. J Gen Intern Med.

[71] Stoddard N, Grewal M, Lavoie N, Krishna A (2021). Fatal *Clostridium septicum* myonecrosis from gastric perforation: a case report. Anaerobe.

[72] Saiyed A, Datta D (2020). A middle-aged woman with hematochezia, hypotension, and leg cramps. Chest.

[73] Slezak M, Smolar M, Drobna Saniova B, Hosala M, Miklusica J (2022). *Clostridium septicum* foot gangrene associated with colorectal cancer. Neuro Endocrinol Lett.

[74] Rimawi BH, Graybill W, Pierce JY, Kohler M, Eriksson EA (2014). Necrotizing fasciitis and toxic shock syndrome from *Clostridium septicum* following a term cesarean delivery. Case Rep Obstet Gynecol.

[75] Zurmeyer S, Fotopoulou C, Braicu E, Schlichting U, Sehouli J (2013). *Clostridium septicum* can cause distant myonecrosis in patients with ovarian cancer. Anticancer Res.

[76] Barbour SA, King W (2003). The safe and effective use of allograft tissue--an update. Am J Sports Med.

[77] Jing HD, Li L, Tian JY, Jiang DP (2021). *Clostridium septicum*-induced gangrene in the right lower extremity complicating pneumatosis in the right ventricle and the pulmonary artery and occlusion of right femoral artery: a case report. BMC Infect Dis.

[78] Winter E, Dommke A, Bongers-Binder S, Eiring P, Weise K (1998). Exogenously acquired *Clostridium septicum* gas gangrene--a case report. Swiss Surg.

[79] Chin RL, Martinez R, Garmel G (1993). Gas gangrene from subcutaneous insulin administration. Am J Emerg Med.

[80] Kirchweger P, Wundsam H, Bosse F, Fritz A, Kratzer T (2022). Systematic literature review and meta-analysis of *Clostridium septicum* aortitis. J Vasc Surg.

[81] Sanchez-Vicente JL, Contreras-Díaz M, López-Herrero F, Martínez-Borrego A, Galván-Ledesma A (2022). Clostridium septicum endogenous endophthalmitis as the initial manifestation of colorectal carcinoma: clinical case report and literature review. Ocul Immunol Inflamm.

[82] Manwani B, Xu Y, El Sahly HM (2020). Hepatic abscesses due to *Clostridium septicum* infection and its association with colonic adenocarcinoma: a case report and literature review. Clin J Gastroenterol.

[83] Macha K, Giede-Jeppe A, Lücking H, Coras R, Huttner HB (2016). Ischaemic stroke and *Clostridium septicum* sepsis and meningitis in a patient with occult colon carcinoma - a case report and review of the literature. BMC Neurol.

[84] Extracorporeal Life Support Organization (2017). ELSO Guidelines for Cardiopulmonary Extracorporeal Life Support. https://www.elso.org/portals/0/elso%20guidelines%20general%20all%20ecls%20version%201_4.pdf.

